# How offshore wind could become economically attractive in low-resource regions like Indonesia

**DOI:** 10.1016/j.isci.2022.104945

**Published:** 2022-08-16

**Authors:** Jannis Langer, Sergio Simanjuntak, Stefan Pfenninger, Antonio Jarquin Laguna, George Lavidas, Henk Polinder, Jaco Quist, Harkunti Pertiwi Rahayu, Kornelis Blok

**Affiliations:** 1 Faculty of Technology, Policy and Management, Department of Engineering Systems and Services, Delft University of Technology, Jaffalaan 5, 2628 BX Delft, the Netherlands; 2 Faculty of Mechanical, Maritime and Materials Engineering, Department of Maritime and Transport Technology, Delft University of Technology, Leeghwaterstraat 17, 2628 CA Delft, the Netherlands; 3 Faculty of Civil Engineering and Geosciences, Department of Hydraulic Engineering, Delft University of Technology, Stevinweg 1, 2628 CN Delft, the Netherlands; 4 Institute Technology of Bandung, School of Architecture, Planning and Policy Development, Jl Ganesha no 10, Bandung 40132, Indonesia

**Keywords:** Energy resources, Energy policy, Energy management, Energy modeling

## Abstract

The current focus of offshore wind industry and academia lies on regions with strong winds, neglecting areas with mild resources. Photovoltaics' cost reductions have shown that even mild resources can be harnessed economically, especially where electricity prices are high. Here, we study the technical and economic potential of offshore wind power in Indonesia as an example of mild-resource areas, using bias-corrected ERA5 data, turbine-specific power curves, and a detailed cost model. We show that low-wind-speed turbines could produce up to 6,816 TWh/year, which is 25 times Indonesia’s electricity generation in 2018 and 3 times the projected 2050 generation, and up to 166 PWh/year globally. Although not yet competitive against current offshore turbines, low-wind turbines could become a crucial piece of the global climate mitigation effort in regions with vast marine areas and high electricity prices. As low-wind-speed turbines are not yet on the market, we recommend prioritizing their development.

## Highlights

• We challenge the notion of mild wind resources being uneconomic in low-wind regions
• Onshore turbines modified for offshore use supply up to 6,816 TWh/year in Indonesia
• Interesting use cases in regions with vast marine areas and high electricity prices
• Mild wind power could be profitable even far offshore without visual impact

## Graphical abstract

**Figure:**
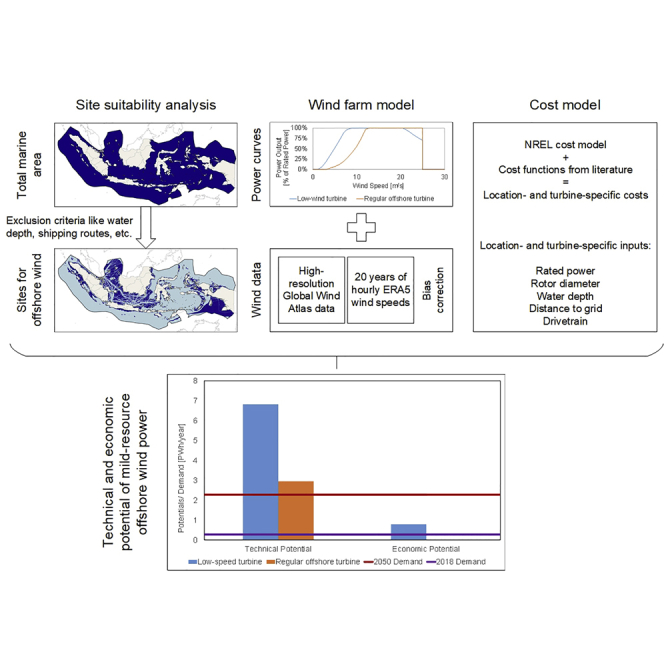

## Introduction

Indonesia is known for many things, but strong winds are not one of them. Compared with high-resource countries like Denmark and the UK with average 100 m wind speeds of 8.5 m/s and higher, Indonesia’s average is less than half, at 4 m/s. Indeed, Indonesia is among the most wind-poor countries globally on average (Wind Energy, 2021). Consequently, wind energy is currently not at the center of Indonesia’s energy transition (Presiden Republik Indonesia, 2017). However, mild renewable resources can still be harnessed economically, either via cost reductions, as has been shown by examples like photovoltaics in Finland (IEA PVPS, 2019), or via high local electricity prices (Musial et al., 2016), e.g., in rural and remote areas worldwide (Seungtaek et al., 2020).

In practice, offshore wind power is becoming an increasingly exclusive technology for regions with high wind resources, whereas low-resource countries like Indonesia remain sidelined. To reduce electricity generation costs (Shields et al., 2021), manufacturers focus on releasing larger and larger turbines (Fragoso Rodrigues, 2016) designed explicitly for high-resource locations. In contrast, we could not find offshore wind turbines on the market designed for mild resources (The Wind Power, 2021a). Current research on wind power potential commonly excludes mild resources using wind speed thresholds, assuming limited economic viability there (Deng et al., 2015; Hundleby and Freeman, 2017; Musial et al., 2016; Schallenberg-Rodríguez and García Montesdeoca, 2018; Vinhoza and Schaeffer, 2021). Studies including mild resources (Bosch, 2018; Caglayan et al., 2019) found that low-capacity turbines are preferable in low-wind-speed regions but excluded local electricity tariffs and did not discuss mild areas specifically. As part of the “LowWind Project” at DTU Wind (Madsen, 2021), a hypothetical, low-specific-power, low-cut-out-wind-speed turbine is studied, but only for North and Central Europe and again not for low-resource regions (Swisher et al., 2022). For Indonesia, past studies (Bosch et al., 2018; Deng et al., 2015; Gernaat et al., 2014; Royal Dutch Shell, 2020; Simanjuntak, 2021; Vidinopoulos et al., 2020) suggested the offshore wind potential may reach up to 14,722 TWh (Gernaat et al., 2014), implying that mild wind resources could significantly contribute to Indonesia’s energy transition. However, these studies used low-resolution wind data (Deng et al., 2015; Gernaat et al., 2014), one type of turbine (Deng et al., 2015; Simanjuntak, 2021), or excluded local electricity tariffs (Bosch et al., 2018; Deng et al., 2015; Gernaat et al., 2014).

Current studies may not capture the impact of detailed orography on local wind profiles and may select turbines unfit for the local conditions. Moreover, comparing the cost of energy technologies without considering local electricity tariffs disregard technologies that are comparatively more expensive but still economically viable in regions with sufficiently high tariffs. Furthermore, wind turbines designed for low wind speeds, e.g., Swisher et al.’s (2022), have not yet been studied for mild-resource regions, so the impact of such a technology for power system decarbonization at these currently excluded regions is still unknown.

To address these shortcomings, we study the technical and economic potential of offshore wind in mild-resource regions, with Indonesia as our representative case. It is important to note that in this study we assess the technical and economic potentials separately, which do not cover the overall techno-economic potential as presented in existing studies (Caglayan et al., 2019; Mattar and Guzmán-Ibarra, 2017; Peña Sánchez et al., 2021). The focus is on turbines designed for low wind speeds: first, to draw attention to the currently overlooked but considerable potential of mild-resource regions in making a significant contribution to a rapid energy transition and second, to understand the overall offshore wind potential in Indonesia. We use 20 years of hourly ERA5 (Hersbach et al., 2018) wind speed data, bias-corrected with the *Global Wind Atlas (GWA)*, and map suitable sites for offshore wind farms based on exclusion criteria. Besides two offshore turbines, we also study two low-wind-speed onshore turbines assumed to be modified for offshore application. We use turbine-specific power curves and a detailed cost model to calculate the turbines’ technical and economic potential. The technical potential aggregates the annual electricity production of all wind farms mapped across Indonesia, whereas the economic potential only includes wind farms with *Levelized Cost of Electricity (LCOE)* equal to or below the local electricity tariff. Furthermore, we assess the sensitivity of our results to changes in site selection criteria and model parameters and show how a carbon tax could boost the technology’s economic potential. We now discuss these aspects in turn.

## Results

### Technical potential of offshore wind in Indonesia

**Figure 1:**
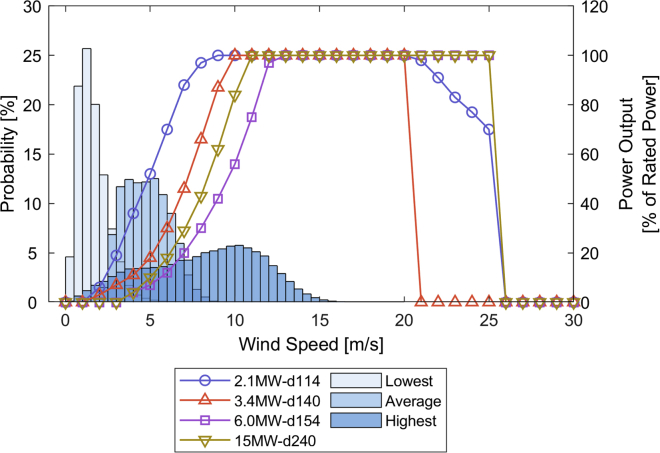
Comparison of representative wind profiles at 100 m height in Indonesia with the four used power curves (normalized to rated power) (The Wind Power, 2021a) The three histograms refer to the wind profiles with the lowest and highest average wind speed and an average profile of all wind farms across Indonesia (DTU Wind Energy et al., n.d.). For clarity, the wind profiles at 150 m hub height relating to the 15MW-d240 are not shown here.

First, we quantify the technical potential by selecting wind turbine model power curves, quantifying the available area for them through geospatial analysis, and combining the two to compute aggregate total wind potentials for Indonesia. We consider four different turbines, for which we use the labeling terminology *[rated power]MW-d[rotor diameter]* for the remainder of the paper. The *2.1MW-d114* and *3.4MW-d140* are onshore turbines designed for mild wind resources and are assumed to be modified for offshore application (see STAR Methods section). The *6.0MW-d154* and *15MW-d240* are offshore turbines that reflect the current state and outlook of the industry. The average capacity factors vary significantly among the turbines, with 35% for the *2.1MW-d114*, 20% for the *3.4MW-d140*, 9% for the *6.0MW-d154*, and 15% for the *15MW-d240*. These capacity factors are below the average factor of 43% from existing offshore wind farms (International Renewable Energy Agency, n.d.). However, the highest capacity factors are 60% for the *2.1MW-d114* and 43% for the *15MW-d240*, which are competitive to the average values expected in 2050 (DNV, 2021; International Renewable Energy Agency, n.d.). The differences in wind profiles and turbines (see Figure 1) cause the wide range of capacity factors. The average wind speed in Indonesia virtually never exceeds 10 m/s. Moreover, turbines with high cut-in and rated wind speed, like the *6.0MW-d154*, rarely operate at rated power. Although the *15MW-d240* shows a better technical performance than the *6.0MW-d154*, it cannot compete with the two modified low-wind-speed turbines. This underscores that current offshore turbines are unsuitable for mild resource regions and that expected future developments in turbine upsizing might not fully address this issue. To better capture mild wind resources, offshore turbines would need a combination of low cut-in and rated wind speed, e.g. the *2.1MW-d114* with 1.5 m/s and 9 m/s, respectively. In Figure 1, the *2.1MW-d114* operates almost continuously at partial load with the average wind profile and at sustained full load in high-resource locations.

Next, we need to place these turbines in feasible locations. Table 1 summarizes the criteria used for site selection and their impact on the excluded marine area and potential. Water depth is the most restrictive criterion despite choosing a threshold depth of 1,000 m, which implies the use of floating turbines. If we restrict the threshold to 55 m depth, i.e., excluding floating turbines, 71% of the total marine area would be removed. Visual impact and shipping routes are not as restrictive, showing that offshore wind power might only have a limited effect on other sectors like tourism, real estate, and shipping. With all exclusion criteria in place, 1.3 million km^2^ of marine area are available for 2.1–3.4 TW of offshore wind capacity.

**Table 1:** Impact of exclusion criteria on marine area and technical potential

Exclusion criterion	Excluded area [km^2^]	Percentage of the total area [%]	Excluded technical potential [GW]
Water depth	3,492,734	58% (71%^a^)	5,588–9,081
Data availability GWA	935,947	16%	1,498–2,433
Visual impact	660,764	11%	1,057–1,718
Shipping routes	581,730	10%	931–1,512
Conservation zones	254,405	4%	407–661
Subsea cables	114,128	2%	183–297
**All criteria combined**	**4,691,716**	**78%**	**7,507–12,198**

The percentage of excluded area foots on the total marine area of 6,020,917 km^2^ within Indonesia’s Exclusive Economic Zone (EEZ). “Data availability GWA” refers to the areas of Indonesia’s EEZ not covered in the Global Wind Atlas (GWA), which is used for bias-correction of the ERA5 wind speed data. The excluded technical potential is based on the range of capacity densities of the studied turbines. The excluded area and technical potential per criterion do not add up to the values in “All criteria combined” because some layers overlap.

a Excluded area rises to 71% if limiting to 55 m water depth, which would exclude floating turbines.

Combining wind turbines and areas and modeling their generation (see STAR Methods), we estimate the technical potential in terms of annual electricity production. We find that low-wind-speed offshore turbines could produce much more electricity in Indonesia with 6,816 TWh/year than currently available offshore turbines like the *6.0MW-d154* with 2,946 TWh/year. This range could cover Indonesia’s electricity generation in 2018 (Badan Pusat Statistik, 2020) 11–25 times and the projected generation in 2050 (Presiden Republik Indonesia, 2017) 1.3–3 times. In relation to Indonesia’s total *Exclusive Economic Zone (EEZ)*, the production density is up to 1.1 GWh/year/km^2^. If this density is applied to the global EEZ for an order-of-magnitude estimation, the global technical potential would be 166 PWh/year or 7 times the global electricity consumption in 2019 (International Energy Agency, 2022). Therefore, low-wind-speed offshore turbines could have a significant impact on the global energy transition.

Our potentials deviate from the ones in literature. The Royal Dutch Shell’s database (Royal Dutch Shell, 2020) gives an offshore wind potential of 3,937 TWh using a minimum wind speed of 8 m/s as described in the study underlying the database (Deng et al., 2015). With such a threshold, our technical potentials would be much lower with 2.6–3.2 TWh, which could be explained by the (1) low resolution of the input data, (2) higher capacity density of 7 MW/km^2^, and (3) power generation function in Deng et al. (2015). Bosch’s (2018) potential of 8,300 TWh/year may be larger than ours due to the (1) higher availability factor, (2) exclusion of transmission losses, and (3) less restrictive site selection. The differences across studies show the importance of transparency about the assumptions and their impact on results.

### Economic potential of offshore wind in Indonesia

**Figure 2:**
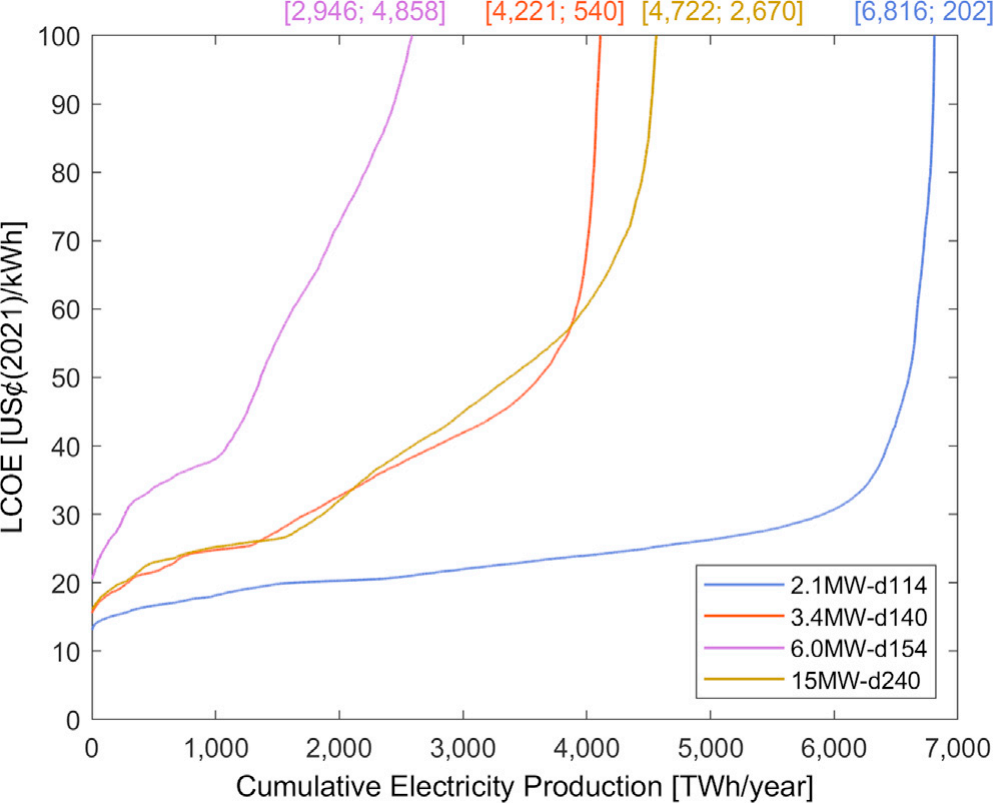
Supply curves for each turbine The y axis is limited to 100 US¢(2021)/kWh to improve readability of the plot. The endpoints of the plots are indicated at the top of the chart in [TWh/year; US¢(2021)/kWh].

For the economic potential, we calculated the LCOE for each wind farm, compared them with the local electricity tariff, and aggregated the annual electricity production of all farms with LCOE lower than or equal to the tariff. Figure 2 shows the supply curves per turbine. The *2.1MW-d114* performs the best economically and could produce 1,626 TWh/year at an LCOE below 20 US¢(2021)/kWh. All other turbines show steeply increasing LCOE due to the comparatively low electricity production. The LCOEs in Figure 2 are far higher than the average LCOE of 8–13 US¢(2018)/kWh observed in practice (International Renewable Energy Agency, 2021, n.d.). Recent wind farms benefitted from deployment in high-resource areas and cost reductions via turbine upsizing (Shields et al., 2021), so it is unclear whether such cost reduction rates are feasible for low-wind-speed, low-capacity wind turbines in mild regions. Nonetheless, we believe that the further development of such turbines could lead to cost reductions and thus improve their economic competitiveness.

**Figure 3:**
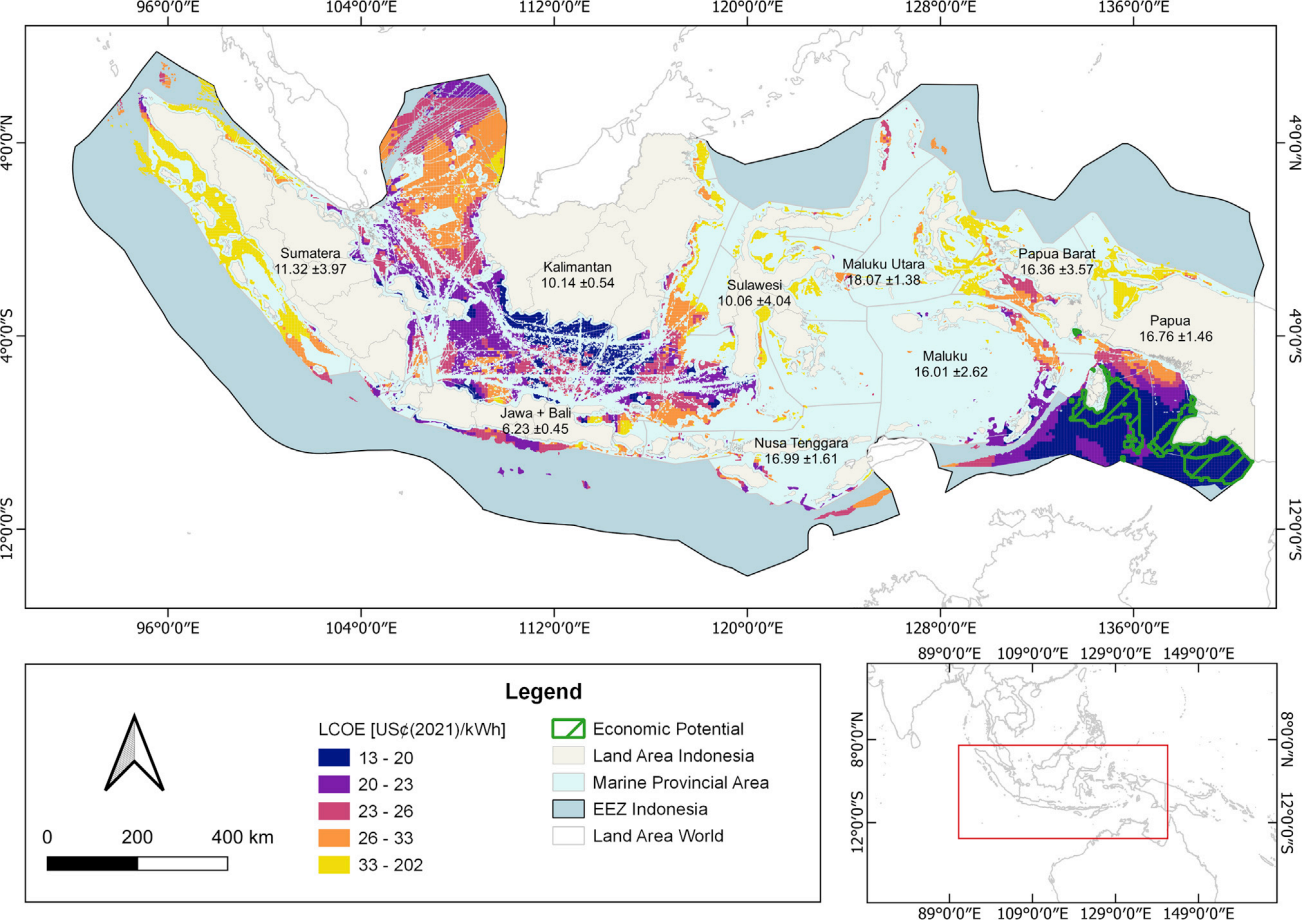
Offshore wind farms in Indonesia and their LCOE for the 2.1MW-d114 Wind farms with economic potential are framed in green. The LCOEs in the legend are scaled and colored by quartiles. The average local electricity tariff and its SD are shown in US¢(2021)/kWh for each island group.

Figure 3 visualizes the wind farms' location and LCOE for the *2.1MW-d114*. LCOE are below 20 US¢(2021)/kWh on Papua, Maluku, and the southern part of Kalimantan. Between the islands of Java and Kalimantan, the impact of shipping routes is clearly visible. Especially at the harbor in Surabaya on Java, many ships head to and from Indonesia’s islands and therefore necessitate the careful planning of offshore wind farms.

Considering the current local electricity tariff, only wind farms on Papua, Papua Barat, and Maluku bear economic potential, outlined in green in the figure. The total economic potential varies significantly across turbines and reaches 784 TWh/year for the *2.1MW-d114*, 22 TWh/year for the *3.4MW-d140*, 0 TWh/year for the *6.0MW-d154*, and 5.6 TWh/year for the *15MW-d240*. The turbines with economic potential could cover the local electricity generation of 2.4 TWh in 2018 (Badan Pusat Statistik, 2020) 2.3–327 times. Hence, low-wind-speed turbines could still be economically viable, as the limited competitiveness against current offshore turbines is compensated by high electricity tariffs.

We show that 100% renewable electricity could be economically feasible in three aforementioned provinces, at least from a resource perspective. Then again, only a tiny fraction of the economic potential could be materialized in practice due to the low local electricity demand in these rural areas. High-demand, low-tariff regions like Java and Sumatera are not economically feasible, at least without further policy support as we show in the following section.

**Figure 4:**
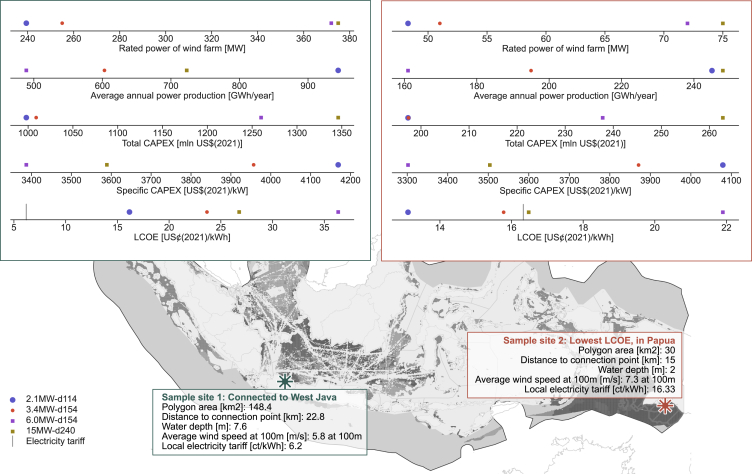
Technical and economic results per turbine at two sites, one close to Java with high electricity demand and the one with the lowest LCOE on Papua

Figure 4 presents the results of two wind farms, one close to Java with high electricity demand and the one with the lowest LCOE on Papua. On Papua, two of the four turbines are economically feasible against the local tariff of 16.33 US¢(2021)/kWh. Meanwhile, none of the turbines bear economic potential on Java, despite a just slightly higher LCOE. The specific CAPEX of 3,302–4,169 US$(2021)/kW harmonize with the values found in literature (Dewan Energi Nasional, 2017; Stehly et al., 2020). The cost reductions from turbine upsizing in Figure 4 align with experts' expectations (Wiser et al., 2016) and the manufacturers' ambitions to scale up their turbine ratings (Shields et al., 2021). For the *6.0MW-d154*, the relative cost savings are outweighed by its limited electricity production on both Java and Papua. The installation cost and OPEX are far higher for the *2.1MW-d114* than for the other turbines due to its small capacity density and increased demand for maintenance. With 114 turbines at sample site 1 and 23 turbines at sample site 2, the installation and maintenance processes are more time- and labor-intensive. Due to the high productivity of the *2.1MW-d114*, we expect faster fatigue of system components and thus more frequent maintenance, overhaul, and reparation activities, which we account for using a kWh-based OPEX component.

The estimates presented here should be considered first-of-a-kind figures. The cost of the first installations is likely to be significantly higher, given that in Indonesia, the necessary infrastructure and equipment to install, operate, and maintain offshore wind farms does not exist yet, and collaboration with experienced, international partners might be required. Therefore, costs are highly uncertain, and our results only serve as indicative projections. Furthermore, further investigations would be necessary to ensure that the sites in Figure 4 are accessible for installation vessels, given the water depth of only 2.5 and 7.6 m. We turn to the sensitivity of model results to uncertain assumptions next.

### Sensitivity to site selection criteria and a carbon tax

**Figure 5:**
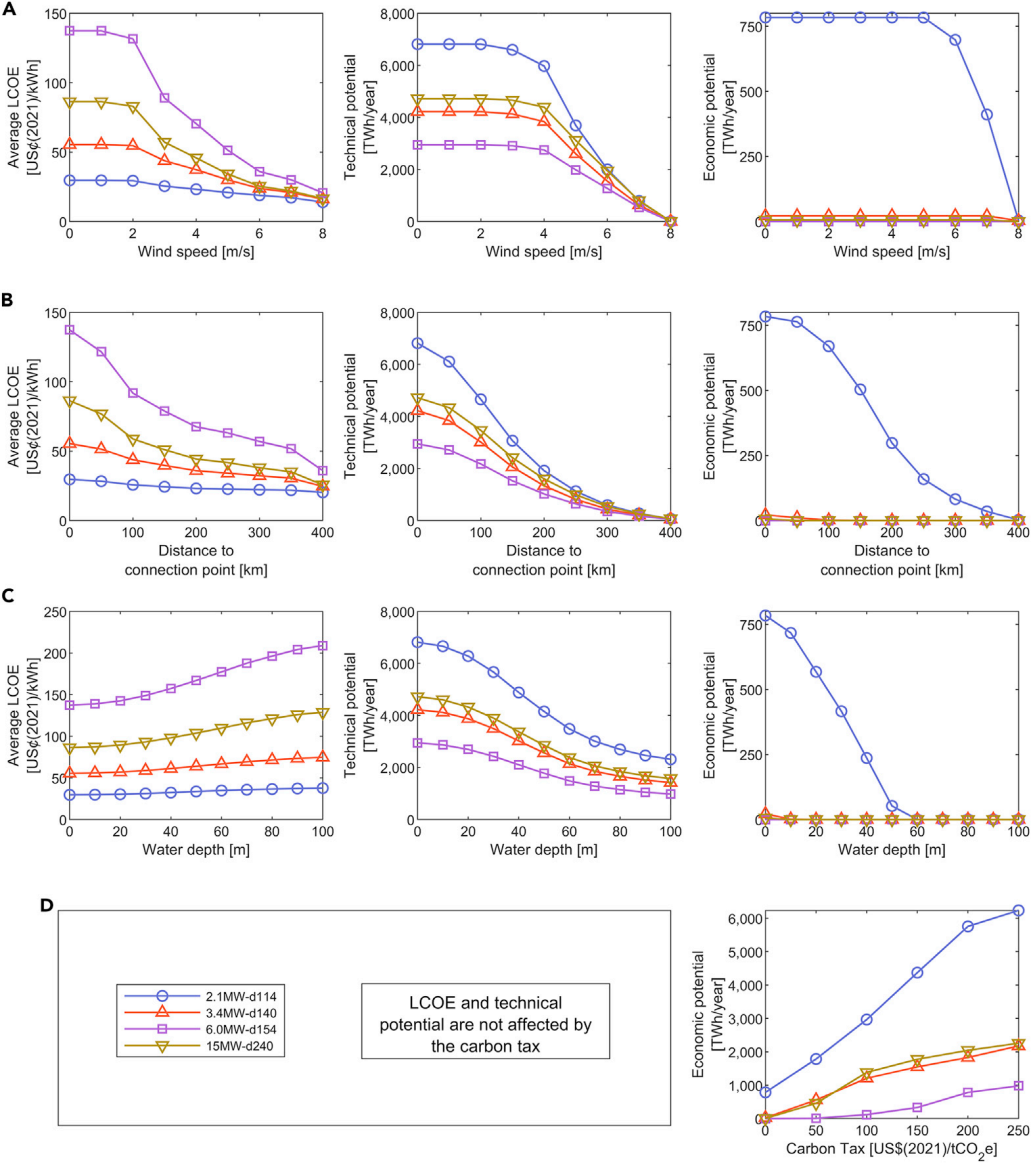
The impact of input thresholds on LCOE, technical potential, and economic potential per turbine The studied thresholds are (A) average wind speed, (B) distance to the onshore connection point, and (C) water depth. The x-axis in (C) is limited to 100 m to better show the graphs, especially at the transition from fixed-bottom to floating turbines at 55 m. Moreover, the impact of (D) a carbon tax on the economic potential per turbine is shown.

This section elucidates the impact of site selection criteria and a carbon tax on the technical and economic potential as well as on the average LCOE per turbine. Figure 5A shows that the LCOE of the *2.1MW-d114* is the least sensitive to changes in minimum average wind speed, whereas the LCOE of the *6.0MW-d154* is the most sensitive. A threshold below 4 m/s is quite ineffective for the technical and economic potential due to the limited power production at such speeds. However, at thresholds above 4 m/s, the potentials decline drastically. Therefore, we argue that thresholds of 7 m/s and higher as used in literature (Deng et al., 2015; Obane et al., 2021; Vinhoza and Schaeffer, 2021) might be too restrictive. Instead, we recommend a threshold of 4 m/s as already done by Peña Sánchez et al. (2021).

As shown in Figure 5B, there are still significant potentials at distances to the onshore connection beyond 100 km. Due to the Sunda Shelf and Sahul Shelf, the waters remain shallow in large parts of Indonesia, even far offshore. These shelves also explain why the average LCOE continuously decreases with distance. Far offshore, wind speeds are higher, and the increased power production makes up for the increased transmission costs and losses. Then again, the decline might not be as steep with distance-dependent cost functions for installation and maintenance, which was not possible due to a lack of data. Because visual impact is an important factor for the social acceptance of wind power (Kim et al., 2013; Schallenberg-Rodríguez and García Montesdeoca, 2018; Vinhoza and Schaeffer, 2021), stricter distance restrictions could have been deployed with limited technical and economic implications, which is a positive outcome of our study.

Figure 5C shows the negative impact of water depth on LCOE due to steeply increasing offshore structure costs. The technical potential is almost equally distributed among fixed-bottom turbines, floating turbines at depths above 100 m, and floating turbines up to a depth of 1,000 m. This shows the interesting geographical contrasts in Indonesia, as there are not only the aforementioned continental shelves with shallow waters but also large deep-sea regions with depths below 7,000 m, like the Banda Sea (GEBCO Compilation Group, 2020). However, floating turbines can probably not harness mild wind resources economically in the near future, as none of our floating wind farms bear economic potential. Nonetheless, we recommend the technology’s re-evaluation, given its continued development.

Figure 5D illustrates the change in economic potential if a carbon tax was added to the current electricity tariffs as computed in Table S3. The curves indicate an S-shaped increase of economic potential with convergence toward the technical potential. All studied turbines show a noticeable rise of economic potential at tax rates below 100 US$(2021)/tCO_2_e, except for the *6.0MW-d154*. This shows that turbines unsuitable for mild wind conditions would not be economically attractive even with strong policy support.

With sufficiently high carbon tax rates, wind power could hold a more prominent role in Indonesia than currently envisioned (Presiden Republik Indonesia, 2017), from a niche solution for rural areas to a key option for nationwide power system decarbonization. At 100 US$(2021)/tCO_2_e, up to 2,965 TWh/year become economically feasible, now also on Sulawesi and Kalimantan. On Java and Sumatera, offshore wind becomes attractive at 150 US$(2021)/tCO_2_e, leading to a total economic potential of up to 4,371 TWh/year. Lifting offshore wind’s economic viability on these islands is important, as electricity demand is much higher there than in the rural east. If restricted by demand, the economic potential grows from 2.4 TWh/year without a carbon tax to 34 TWh/year with a tax of 100 US$(2021)/tCO_2_e and to 153 TWh/year with a tax of 150 US$(2021)/tCO_2_e. These potentials would cover 1%, 12%, and 55% of the electricity generation in 2018 (Badan Pusat Statistik, 2020), respectively. Although a tax of 150 US$(2021)/tCO_2_e is similar to the ones in Sweden, Switzerland, and Liechtenstein (The World Bank, 2021), it is significantly higher than Indonesia’s carbon tax of 2.1 US$(2021)/tCO_2_e effective from April 2022 (Suroyo and Munthe, 2021). Therefore, Indonesia’s policymakers would have to introduce more ambitious tax rates to materialize offshore wind’s economic potential beyond the rural east.

### Sensitivity to wind farm and cost model parameters

**Figure 6:**
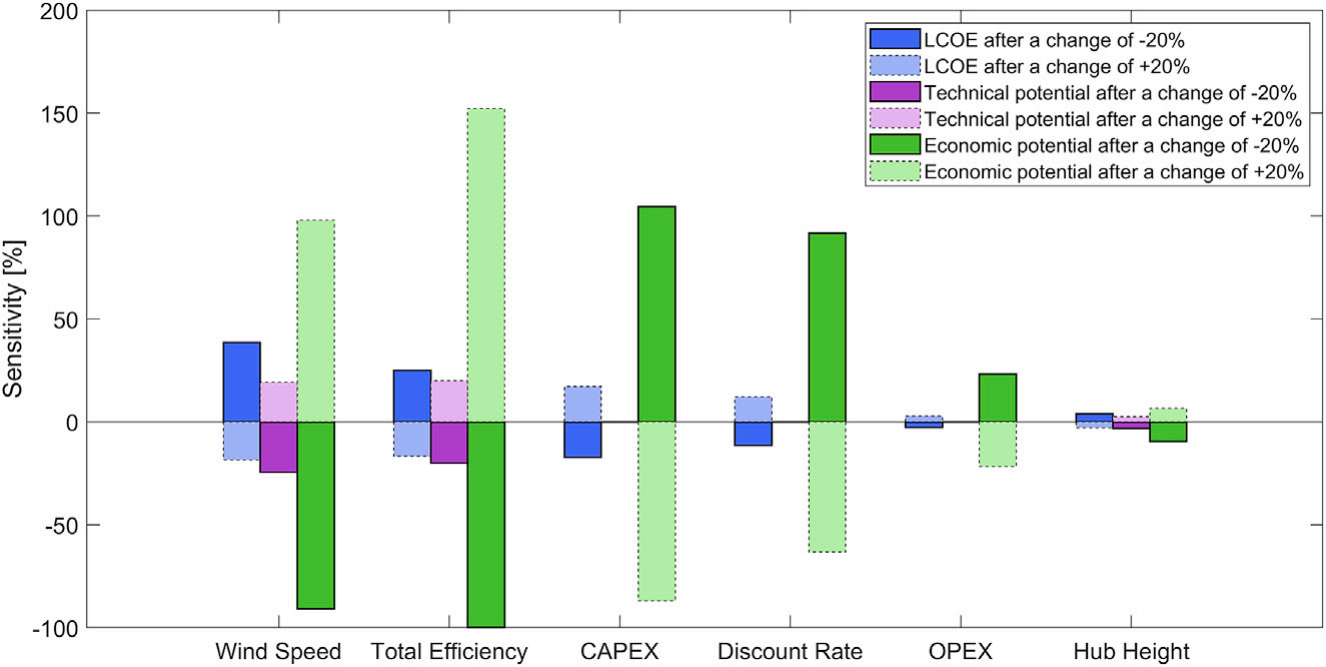
Sensitivity of average LCOE, technical potential, and economic potential of the 2.1MW-d114 to changes in model parameters by ±20%

In the previous sections, *2.1MW-d114* showed the best technical and economic performance, which is why this section solely focuses on this turbine. Figure 6 shows the sensitivity of our results to changes in six model parameters. The technical potential is the least sensitive, with wind speed being the most impactful. Therefore, the robustness of the technical potential could be effectively increased with more accurate wind speed data from measurement and hindcast campaigns at selected areas and hub height. Such data could also validate the wind profiles of our study and offer a better understanding of the long-term wind characteristics. The hub height has a small impact on the technical potential and on cost-related parameters none at all.

The economic potential is by far the most sensitive output. Especially the total efficiency (i.e., availability factor, transmission and wake losses) has a high impact, which shows that more detailed studies on its components are necessary. Since such a study might not be computationally feasible for the entire country, our study could be useful to detect interesting sites suitable for a more localized analysis. The impact of the total efficiency could also indicate that even low-wind-speed, low-capacity wind turbines might have to be upsized eventually to decrease wake losses and to save costs from having less turbines within a wind farm.

Regarding CAPEX and discount rate, Figure 6 shows that mild offshore power could have a substantially higher economic potential when the technology is more developed. Technological learning could reduce CAPEX and investment risks, with potentially positive impacts on interest rates for project finance. The OPEX has a relatively low impact on LCOE and economic potential, thus curbing the severity of OPEX-related limitations of our models. Nonetheless, given Indonesia’s limited infrastructure for offshore wind projects today, future research should design and optimize possible O&M strategies considering infrastructure improvements. The economic potential barely changes with varying hub heights due to the neutralizing effects of power production and tower costs.

## Discussion

This paper shows that low-wind-speed turbines are interesting for mild-resource regions like Indonesia. With a technical potential of up to 6,816 TWh/year, such turbines could perform substantially better in Indonesia than currently available and envisioned offshore turbines. Although low-wind-speed offshore turbines would not yet be competitive against existing wind farms in high-resource regions, they could still be attractive in regions with high electricity tariffs, like in rural Indonesia with an economic potential of 784 TWh/year. This insight holds a global relevance, as much higher tariffs than in Indonesia can be found in parts of USA, Brazil, Australia, and India, amongst others (Seungtaek et al., 2020). Policy support, for example via a carbon tax of 150 US$(2021)/tCO_2_e, would vastly extend the economic potential in Indonesia to 4,371 TWh/year to more developed regions with lower tariffs but much higher electricity demand.

**Figure 7:**
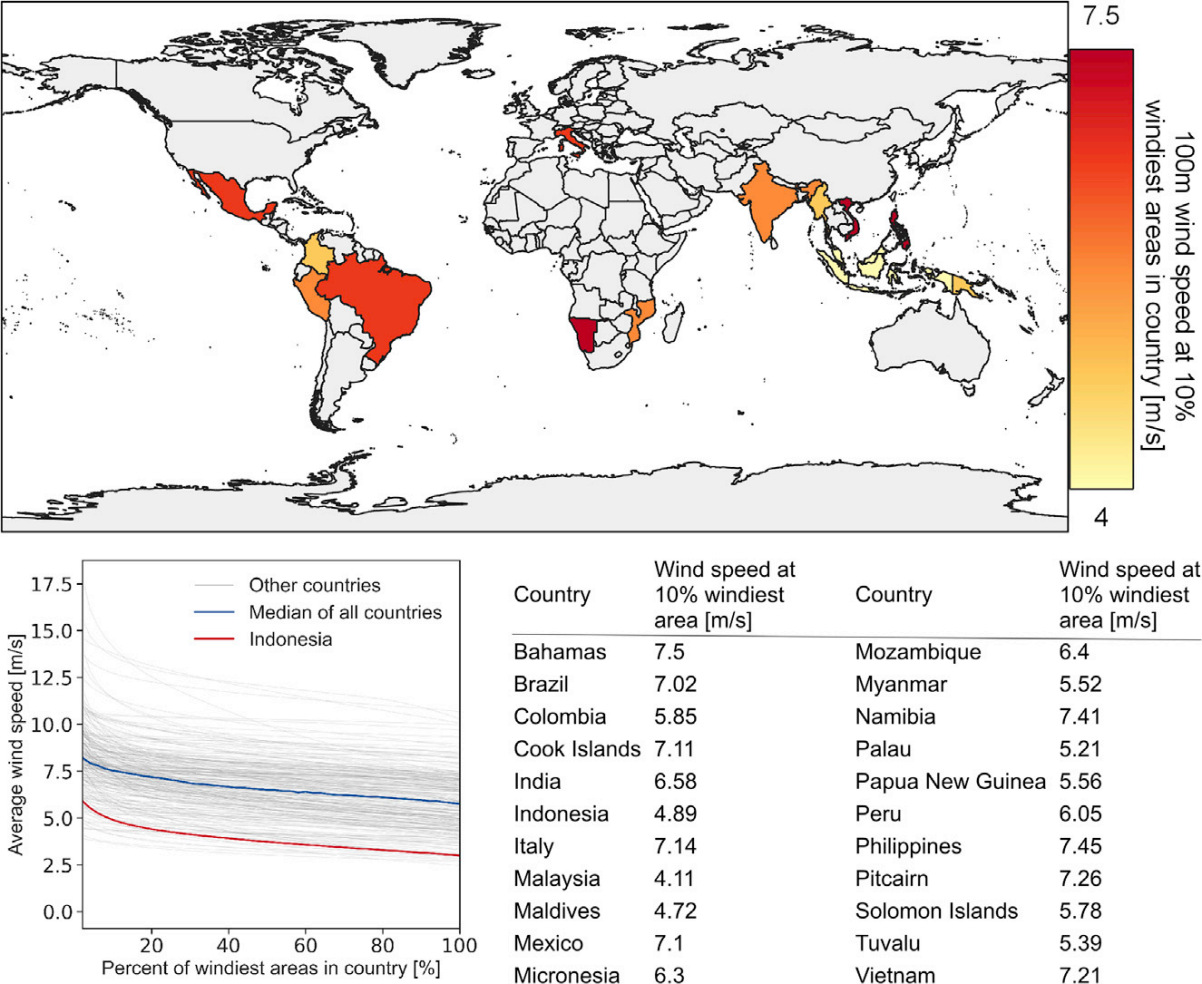
Overview of 22 interesting countries for low-wind-speed offshore turbines The countries were chosen based on a 100m wind speed at the 10% windiest sites (Wind Energy, 2021) of less than 7.5 m/s and an offshore EEZ area of at least 500,000 km^2^. Nonetheless, there might also be high potentials in countries with smaller EEZ or mild-resource spots in otherwise high-resource regions. Note that the term “10% windiest sites” is adopted from GWA (ibid.) and refers to the 90^th^ percentile of wind speeds in a country or region.

However, low-wind-speed offshore turbines are not yet on the market and need to be developed from scratch or by modifying existing low-wind-speed onshore turbines for offshore use. Such turbines could then be a highly interesting technology not only for Indonesia but also for many other regions with mild wind resources, vast marine areas, and high electricity tariffs. As shown in Figure 7, not only South-East Asia could be an interesting hub for mild-resource wind power but also South America with high-electricity-demand countries like Brazil, Mexico, Colombia, and Peru. Moreover, there might be vast potentials in India, where offshore wind could supply more than a billion people with electricity.

We conclude that mild offshore wind energy deserves more attention than it currently receives. With the industry’s move toward larger turbines and higher wind speeds, offshore wind energy will gradually become a technology exclusively appropriate for regions with sufficiently high wind resources. However, successful climate change mitigation requires the rapid transition to climate-neutral electricity supply everywhere in the world. With low-wind-speed offshore turbines, manufacturers could tap a new market with a much broader potential user base, whereas decision makers would have another, previously inaccessible, option to decarbonize their energy systems.

To materialize these prospects, much still needs to happen. Given the sensitivity of our results, further research is necessary that validates our findings and further expands upon the methods we used. Future studies could focus on (1) improved modeling of wind farm spacing and wake losses, (2) more detailed operational expenses excluding site-specific strategies, (3) more detailed inclusion of local site conditions, (4) the assessment of dynamic cost developments from wind farm upsizing and technological learning, and using (5) higher fidelity wind datasets that reduce uncertainties associated with low-resolution data, such as not being able to capture rapid wind speed changes and thus leading to overestimation of the resource. With a better understanding of its potential, policy support could make low-wind-speed offshore power an attractive proposition for manufacturers, letting it grow from an unimportant niche to a regionally important piece of the global clean electricity puzzle.

### Limitations of the study

This section discusses the main limitations of this paper’s methods. Regarding our wind farm model, the availability factor *a*_*f*_ depends on the design of the wind farm as well as on the operation and maintenance strategy (Myhr et al., 2014). But due to limited data on service ports and vessel availability, amongst others, we decided to use a simplified general factor of 90% (Deng et al., 2015; Hong and Möller, 2011), which is comparatively conservative (Cevasco et al., 2021; Martinez and Iglesias, 2022).

Moreover, we do not model the inter-array infrastructure in detail but incorporate it in the total electrical efficiency *η*_*elec*_. The inter-array infrastructure costs are included in the cost components “electrical connections” and “marinization” in Table S2.

For transmission lines, we assume straight lines from plant to onshore connection without the ducting of the lines under water. Depending on the complexity of the seabed structure and local metocean conditions, the transmission costs might be considerably higher. Losses from transformers, converters, and others are assumed to be constant, whereas losses in the transmission cables only depend on the distance to shore.

Another limitation is the use of a general turbine spacing of *10d×* 10d (Bosch, 2018) with wake losses of 88% (Bosch et al., 2018; Nagababu et al., 2017). These values are rather conservative compared with spacings (Schallenberg-Rodríguez and García Montesdeoca, 2018) and wake losses in literature (Hong and Möller, 2011; Schallenberg-Rodríguez and García Montesdeoca, 2018). Given Indonesia’s size, it was computationally not feasible to optimize the spacing and wake losses for each wind farm, which could improve the technical and economic results presented here.

Moreover, we use hourly wind speed data and match them to the power curves that are derived for different time intervals, like 10 min. Therefore, the electricity production might vary from the results shown here if these discrepancies would be addressed.

The cost model and surrounding assumptions also come with limitations. First, we exclude costs for the extension of the local power grid unlike other studies (Dicorato et al., 2011; Möller et al., 2012; Myhr et al., 2014).

Second, some site-specific conditions could not be included in the cost model, like the influence of seabed properties on structure costs. Hong and Möller (2011) assumed 40% higher structure costs in China than in Europe due to different seabed properties. We refrained from such general assumptions, as seabed properties vary across Indonesia. On the western side of Indonesia, seabeds consist of sand, silt, mud, and calcareous ooze, whereas the eastern part also contains large areas of siliceous ooze and clay (Badan Geologi, 2010). We also excluded local wave behavior in the cost model, as waves in Indonesia are rather low (Ribal et al., 2020) and within the operational limits of most vessel operators (Lavidas et al., 2018).

Third, our cost model can only provide a rough estimation of the turbine- and location-specific costs despite the modifications described earlier. Cost components like OPEX and installation costs are simplified and exclude aspects like proximity to service harbor, vessel cost, as well as personnel (Bosch et al., 2019; Myhr et al., 2014). Moreover, our cost model does not reflect the cost developments from (1) wind farm upsizing and (2) technological learning. Regarding (1), we assume no cost savings due to wind farm upsizing (Dicorato et al., 2011; Voormolen et al., 2016). However, we acknowledge the ongoing discussion on this topic and the studies that argue otherwise (Maness et al., 2017; Myhr et al., 2014; Shields et al., 2021). Regarding (2), technological learning could be studied with learning rates by creating implementation scenarios, which is beyond the scope of this paper.

Lastly, cost developments depend on the local policy environment (Voormolen et al., 2016) and commodity prices (Van der Zwaan et al., 2012), amongst others. It is yet unclear how costs will develop in Indonesia, where wind energy is still a nascent technology. Therefore, our cost estimations and their developments must be reassessed when more practical data for Indonesia become available.

Despite the limitations described earlier, we believe that this paper still produces valuable first results, which might spark further, in-depth research in the future.

## STAR★Methods

### Key resources table

**Table:** 

REAGENT or RESOURCE	SOURCE	IDENTIFIER
**Deposited data**		

Code and key output data reported in this paper	NA	https://doi.org/10.4121/19625259
ERA5 hourly data on single levels from 1979 to present - Eastward wind speeds at 100m *u100* - Northward wind speeds at 100m *v100*	(Hersbach et al., 2018)	https://doi.org/10.24381/cds.adbb2d47
Global Wind Atlas data for Indonesia at 100m	(DTU Wind Energy et al., n.d.)	N/A
Conservation zones	(Direktorat Konservasi Kawasan dan Jenis Ikan, 2013; Ministry of Energy and Mineral Resources, 2019a)	N/A
Water depth	GEBCO Compilation Group, 2020)	https://doi.org/10.5285/a29c5465-b138-234d-e053-6c86abc040b9
Shipping routes	(Cerdeiro et al., 2020)	N/A
Subsea cables	(TeleGeography, 2021)	N/A
Wind turbine data	(Gaertner et al., 2020; The Wind Power, 2021a)	N/A

**Software and algorithms**		

NREL’s mass based wind turbine cost model	(Fingersh et al., 2006)	N/A

### Resource availability

#### Lead contact

Further information and requests for resources and materials should be directed to and will be handled by Jannis Langer (j.k.a.langer@tudelft.nl).

#### Materials availability

This study did not generate new unique materials.

### Method details

This section describes the methods to map the wind resources in Indonesia and calculate the technical and economic offshore wind potential. All analysed wind turbines are horizontal-axis machines situated offshore either with a fixed-bottom or floating structure. We included floating turbines in our analysis to reflect the potential of future technologies as in other studies (Bosch et al., 2018; Deng et al., 2015; Schallenberg-Rodríguez and García Montesdeoca, 2018). We acknowledge the current technological and economic barriers of floating turbines. Therefore, even if our analysis yields an economic potential for floating turbines, we do not expect its materialisation in the foreseeable future. Instead, the technology will probably be developed in high-resource regions and only spill over to milder regions once sufficient experience has accumulated.

#### Mapping of suitable sites and wind farm sizing

We use *QGIS 3.16 Hannover* to map suitable sites for offshore wind energy, starting with a base map of Indonesia’s *Exclusive Economic Zone (EEZ)*. We added exclusion layers and their buffers to the base map and removed overlapping areas. In this study, the exclusion layers contain conservation zones, water depth, shipping routes, subsea cables and visual impact (see below table). The output of this step is a shapefile with thousands of polygons suitable for wind farm implementation. We removed polygons smaller than 30 km^2^ to ensure a sufficient wind farm size and to curb computational efforts for subsequent calculations. We divide the remaining polygons into rectangular grid cells with a resolution of 0.125°, and the polygons inside these grid cells represent one wind farm. The subdivision helps to better capture the local wind farm site conditions, like water depth and wind speed, as these values might not be represented adequately if they are averaged over a too large polygon area. Next, the centroids of the gridded polygons are obtained, which are used to store the technical and economic properties of the wind farms, like area and water depth.

**Table:** Exclusion criteria for the mapping of suitable offshore wind farm sites

Exclusion layers [Ref]	Layer type + Resolution	Exclusion criteria	Buffer [m] [Ref]
Conservation zones (Direktorat Konservasi Kawasan dan Jenis Ikan, 2013; Ministry of Energy and Mineral Resources, 2019a)	Vector	–	1,000 (Díaz and Guedes Soares, 2020; Hong and Möller, 2011)
Water depth (GEBCO Compilation Group, 2020)	Raster, 463 m	>1,000 m (Bosch, 2018; Deng et al., 2015)	None (Díaz and Guedes Soares, 2020)
Shipping routes (Cerdeiro et al., 2020)	Raster, 555 m Rescaled to 3 km due to computational limitations	Shipping density <5,000,000 + areas larger than 30.5 km^2^	1,000 (Hong and Möller, 2011)
Subsea cables (TeleGeography, 2021)	Vector	–	1,000 (Bosch et al., 2019)
Visual impact	Vector	<10 km (Kim et al., 2013)	None

The remaining marine areas host the technical offshore wind potential.

#### Creation of bias-corrected wind speed data

We modify the approach from Staffell and Pfenninger (2016) in three ways to obtain 20 years of bias-corrected, spatiotemporally resolved wind speed data across Indonesia. First, we use the newer ERA-5 data instead of MERRA-2 data to benefit from the former’s higher resolution and availability of speeds at 100 m height, which is the default hub height in this study. Second, we do not spatially interpolate to exact wind farm locations but to a finer grid, as detailed below. Third, we bias-correct wind profiles with the *Global Wind Atlas (GWA)* due to a lack of measured data. As of September 2021, there are only two operational wind farms in Indonesia (The Wind Power, 2021b), both being onshore. Wind resource measurement at offshore locations is also unavailable since previous measurement campaigns only took place at onshore locations at heights between 30–50 m (Directorate General of Renewable Energy and Energy Conservation, 2020). This leads to the following procedure:

1. Download 20 years of ERA5 wind speed data at a height of 100 m with a resolution 0.25° and remove outliers.
2. Interpolate linearly between the data points for a finer grid resolution of 0.125°.
3. Bias-correct the wind profiles with GWA data.

#### Download and pre-processing of wind speed data

The setup in the below table is used to download 20 years of ERA5 wind speed data for Indonesia. The timespan was chosen to cover the commonly assumed (Bosch, 2018; McKenna et al., 2014; Nagababu et al., 2017) useful lifetime of a wind farm, but we acknowledge that the timespan could be extended to 25 (Mattar and Guzmán-Ibarra, 2017; Stehly et al., 2020) or 30 years (Schallenberg-Rodríguez and García Montesdeoca, 2018). ERA5 includes both horizontal wind components U=(ux; uy), and both eastward and northward wind speeds must be used to obtain the resulting wind. Outliers are detected with a moving two-week average and replaced via linear interpolation, which affected 0.5% of the total dataset. The dataset is cleaned from outliers while keeping extreme wind speeds caused by rare extreme weather phenomena like tropical cyclones. The output of this step is a cleaned 20-year dataset of hourly wind speed data in a spatial resolution of 0.25° at a height of 100 m.

**Table:** Metadata of the 20 years of hourly ERA5 wind speed data in Indonesia used in this study

Title	Wind Speed Data
Name	ERA5 hourly data on single levels from 1979 to present
Creator	Hersbach et al. (2018) (Hersbach et al., 2018)
Downloaded from	Copernicus Climate Change Service (C3S) Climate Data Store
Web Link	https://cds.climate.copernicus.eu/cdsapp#!/dataset/reanalysis-era5-single-levels?tab = form
Coordinate system	World Geodetic System1984 (WGS84)
Coordinates	92° E to 142° E; 8° N to 13.9° S
Spatial resolution	0.25 × 0.25°
Data type	Point
Retrieved data	Eastward wind speeds at 100m *u100* Northward wind speeds at 100m *v100*
Parameter unit	m/s
Time period	01 January 2001 00:00 to 31 December 2020 23:00
Temporal resolution	1 h

How the data is processed for the technical and economic analysis is described in the following sub sections.

#### Interpolation of wind speed data and bias-correction

As discussed in Staffell and Pfenninger (2016), available reanalysis datasets have a rather low spatial resolution. Therefore, they require bias correction to reflect the impact of the local orography. In this paper, bias correction occurs in two steps. First, the shape of the wind profiles is modified by spatially interpolating between the reanalysis data points as elaborated below and visualised in below figure.

**Figure:**
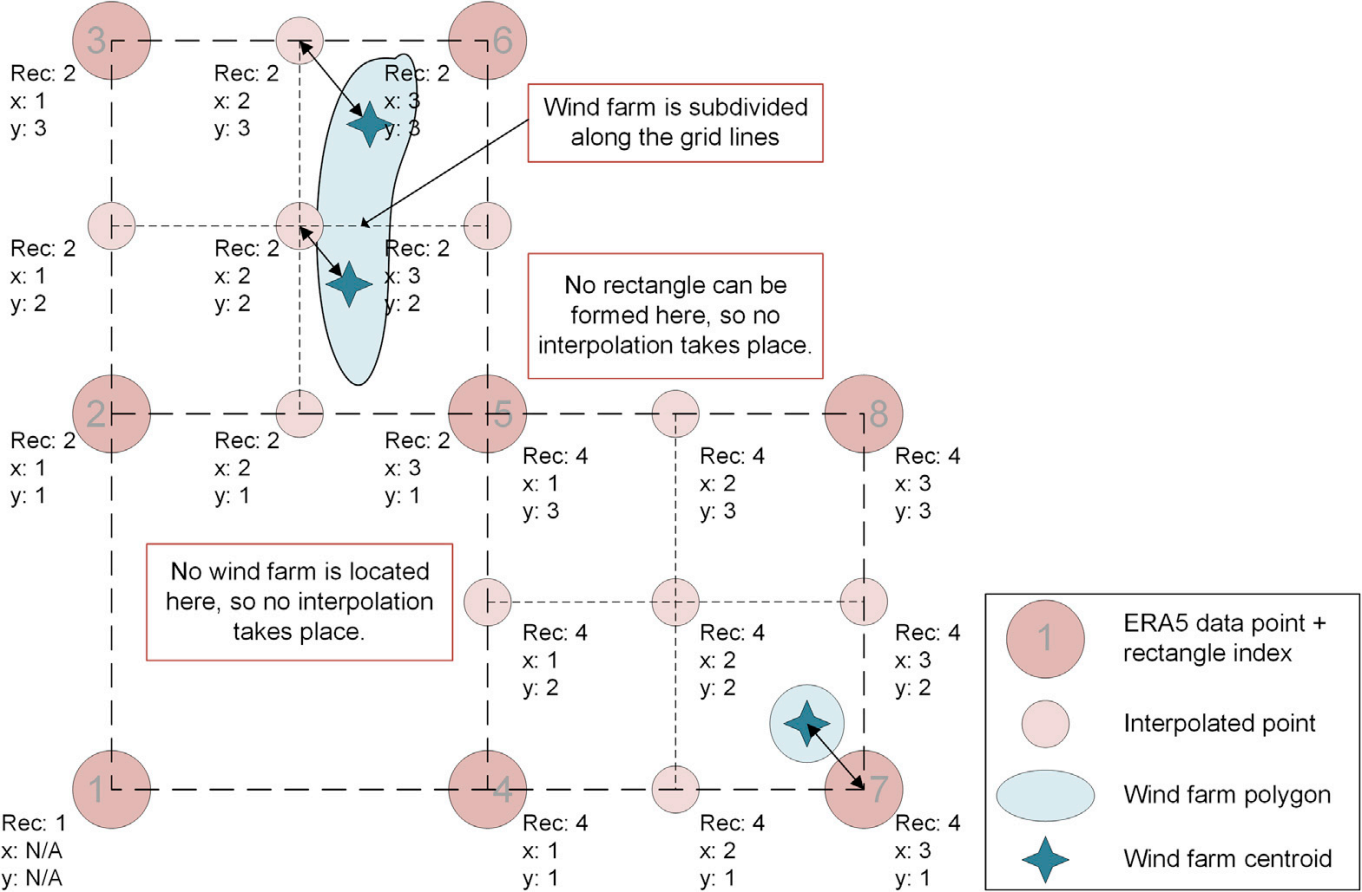
Interpolation of ERA5 wind speed data and indexing convention to connect wind farm centroids with wind speed profiles

In short, we assign each wind farm centroid to its closest point on a finer-meshed grid of 0.125 degree resolution, then linearly interpolate from the native 0.25 degree ERA5 grid to the wind farm grid points where needed (i.e., where a wind farm actually exists). We acknowledge that this approach comes with limitations. The furthest distance between a centroid and a data point is roughly 10 km (The hypotenuse of a triangle with the length of the two legs being 13.9 km/2 = 6.9 km). So in the worst case, two wind farms share one wind profile despite being almost 20 km apart from each other. However, this limitation is addressed with the bias correction using GWA data (see main text). In the following we describe in detail how the interpolation procedure is implemented. First, the coordinates of the ERA5 data points are extracted. For every data point, a numerical code written with *Matlab R2020b* tries to form a square with its neighbours, with the data point being at the bottom left corner. The data point receives a rectangle index, with which the rectangle is identified later. Next, the script checks whether there are any wind farm centroids inside the rectangle. If so, then the wind speed data in the corners of the rectangle are interpolated linearly in a resolution of 0.125° or roughly 14 km for all time steps, resulting in a total of nine wind speed profiles per rectangle. Each of the nine data points receives an x-index and y-index. The interpolated wind profiles and their indices are saved in a separate file. Next, the script loops through all wind farm centroids and adds the three indices of the data point closest to the centroid. With the three indices, the wind farm centroid can be matched with the correct wind profile without compromising the data structure of the involved files for later processing steps. Moreover, computational efforts are reduced, as interpolation is only performed where necessary. Centroids that are assigned to the same data point share one wind profile, therefore the size of the file that stores the wind profiles is limited as well.

Then, the profiles are bias-corrected with a factor based on a high-resolution wind map. We use GWA 3.0 (Wind Energy, 2021), which maps wind speeds with a spatial grid size of 250 m at various heights and uses underlying high-fidelity wind resource hindcast datasets and measuring campaigns for validation, amongst others from Papua New Guinea. There, the average mean absolute bias across three measurement stations was 12% ±10% standard deviation. As Papua New Guinea borders East Indonesia, the bias and thus the wind map are deemed acceptable for this research (DTU Wind Energy et al., n.d.). We follow Bosch et al. (2017) and use a time-invariant, constant correction factor for each wind farm centroid. As the GWA map shows average wind speeds from 2008–2017, the interpolated ERA5 wind profiles are averaged for this period and then compared to the GWA values. The correction factor is then deduced from the deviation of the two averages. For example, if the GWA wind speed at a given centroid is 25% higher than the average interpolated ERA5 wind speed, each value of the wind profile is increased by 25%.

#### Technical and economic analysis of offshore wind power

##### Levelized cost of electricity and choice of turbines

In this sub-section, we summarise our approach for the technical and economic analysis. We calculate the *Levelized Cost of Electricity (LCOE)* per wind farm *c* using Equation (1). The LCOE indicates the necessary electricity tariff to break even with all project costs at the end of the project’s useful lifetime. All costs below are converted to 2021 values with the currency conversion rates in Table S1 (Bureau of Labour Statistics, 2021; Macrotrends, 2021). All assumptions fed into the equations below are shown in below table.

**Table:** General and turbine-specific techno-economic assumptions used in this study to calculate the technical and economic potential of mild offshore wind power in Indonesia

General Assumptions [Ref]					
Discount rate *i* [%]			10 (IESR, 2019; KPMG, 2019)		
Lifetime *N* [years]			20 (Bosch, 2018; McKenna et al., 2014; Nagababu et al., 2017)		
Wake efficiency *η*_*Wake*_ [%]			88 (Bosch et al., 2018; Gebraad et al., 2016; Nagababu et al., 2017)		
Availability factor *a*_*f*_ [%]			90 (Deng et al., 2015; Hong and Möller, 2011)		
Turbine spacing [-]			10*d ×* 10*d* (Bosch, 2018)		

Turbine-specific assumptions (all information from (The Wind Power, 2021a) and (Gaertner et al., 2020))					
Commercial name	SG2.1-114	GW140-3400		SWT-6.0-154	IEA 15 MW RWT
Name in this paper	2.1MW-d114	3.4MW-d140		6.0MW-d154	15MW-d240
Rated power [kW]	2,100	3,400		6,000	15,000
Assumed hub height [m]	100	100		100	150
Cut-in wind speed [m/s]	1.5	2		4	3
Rated wind speed [m/s]	9	10.5		13	10.6
Cut-out wind speed [m/s]	25	20		25	25
Rotor diameter [m]	114	140		154	240
Capacity density [MW/km^2^]	1.6	1.7		2.5	2.6
Wind class	IEC IIA/IIIA/S	IEC IIIA		IEC IA	IEC IB
Onshore/offshore application	onshore	onshore		offshore	offshore
Direct drive	no	no		yes	yes
Ratio of generator capacity to swept area [W/m^2^]	206	221		322	332

The limitations of the assumptions are discussed in the paper.

(Equation 1) $$\begin{array}{l} LCOE_{c}=\frac{CAPEX_{c,0}+\sum_{n=1}^{N}\frac{OPEX_{c,n}}{{\left(1+i\right)}^{n}}}{\sum_{n=1}^{N}\frac{E_{a,c,n}}{{\left(1+i\right)}^{n}}} \end{array}$$

CAPEX and OPEX are capital and operational expenses, respectively, and their costs are explained in the next sub-section. *i* is the discount rate and *n* is the year with lifetime *N* being the maximum value. The annual electricity production *E*_*a*_ is computed with Equation (2) as a function of wind speed *v*, the distance between the wind farm to an onshore connection point *l,* the availability factor *a*_*f*_*,* and the number of wind turbines *H* (Bosch, 2018). *T* relates to the timestep out of *T=8,760 hours/year*.

(Equation 2) $$\begin{array}{l} E_{a,c,n}=\sum_{t=1}^{T}P_{Turb}\left(v_{c,t,n}\right)*H_{c}*\eta_{elec}\left(l_{c}\right)*\eta_{Wake}*a_{f} \end{array}$$

The number of wind turbines *H* in a wind farm is calculated with Equation (3) with the longitudinal and latitudinal turbine spacing *S*, rotor diameter *d,* and wind farm area *A*.

(Equation 3) $$\begin{array}{l} H_{c,Turb}=\frac{A_{c}}{S_{long}*d_{Turb}*S_{lat}*d_{Turb}} \end{array}$$

Furthermore, the electrical losses from the inter-array and transmission infrastructure *η*_*elec*_ are calculated with Equation (4) as a function of distance *l* from wind farm to onshore grid connection point.

(Equation 4) $$\begin{array}{l} with\;\eta_{elec,c}\left(l_{c}\right)=\left\{ \begin{array}{l} \begin{array}{lll} 0.979-1*10^{-6}*l_{c}^{2}-9*10^{-5}*l_{c}, & |\;l_{c}\leq 50\;km & \end{array}\\ \begin{array}{lll} 0.964-8*10^{-5}*l_{c},\; & |l_{c}>50\;km & \end{array} \end{array}\right. \end{array}$$

Depending on the distance, either *High-Voltage Alternating Current (HVAC)* cables at 220 kV or *High-Voltage Direct Current (HVDC)* cables at 320 kV are assumed (Fragoso Rodrigues, 2016). The default hub height *h* in this study is 100 m (Bosch, 2018). If a turbine cannot operate at such height, e.g. due to too long rotor blades, the wind speed *v* is scaled for the alternative height using the power law. The local shear exponent *α* is calculated with Equation (5) and (6) with GWA data at 50 and 100 m height (Nefabas et al., 2021).

(Equation 5) $$\begin{array}{l} v_{x,c,t}=v_{100m,c,t}*{\left(\frac{h_{\pm x}}{h_{100m}}\right)}^{\alpha_{c}} \end{array}$$

(Equation 6) $$\begin{array}{l} \alpha_{c}=\frac{\ln\left(\frac{v_{100m,c}}{v_{50m,c}}\right)}{\ln\left(\frac{h_{100m}}{h_{50m}}\right)} \end{array}$$

We use the power curves *P*_*Turb*_*(v)* of four turbine models from the Wind Power database (2021a) and IEA 15 MW reference turbine (Gaertner et al., 2020) to calculate *E*_*a*_. The latter turbine is included to reflect the trend of the offshore wind industry for increasingly larger turbines with greater rated power and longer rotor blades (Enevoldsen and Xydis, 2019). Instead of their commercial names, we use a standardised terminology of *‘[rated power]MW-d[rotor diameter]*’ to refer to turbines. The turbines are selected to have a variety of rated power, rotor diameter, and cut-in, rated, and cut-out wind speed. The power curves are not smoothened as in other studies (Bosch et al., 2017; Staffell and Pfenninger, 2016) to avoid an overestimation of electricity production, as the smoothened power curves can entail a higher power output at low wind speeds (Bosch et al., 2017; De Tommasi et al., 2010).

Note that all turbines suitable for IEC wind class III are onshore turbines. Since there are currently no IEC wind class III offshore turbines on the market, the power curves of the onshore turbines are used for offshore application. For the sake of the analysis, we argue that the onshore turbines could be modified for offshore use and deployed with an adequately designed support structure and tower to withstand wave loading forces. These requirements are incorporated into the cost model in the next sub-section. For completeness, we also include one offshore turbine to show the technical and economic potential of existing offshore turbines.

##### Cost model for fixed-bottom and floating wind farms

We use the mass-based cost model developed by the *National Renewable Energy Laboratory (NREL)* (Fingersh et al., 2006). CAPEX and OPEX can be calculated based on rotor diameter, hub height, rated power, and drivetrain type. The model found application in academic literature (McKenna et al., 2014; Sliz-Szkliniarz et al., 2019) but also faced criticism. Rinne et al. (2018) rightfully pointed out that the methodology is somewhat outdated given that it was developed in 2006. Updating the cost functions with industrial data is challenging, as almost all project contracts are confidential (Shields et al., 2021; Voormolen et al., 2016). Moreover, using constant system cost factors per rated power (Möller et al., 2012; Schallenberg-Rodríguez and García Montesdeoca, 2018) can lead to inaccurate cost estimations as they exclude location- and turbine-specific influences on cost. Therefore, we propose two modifications to bring NREL’s cost model up to date.

First, we replace the cost functions of some components with more recent functions and values from literature. The offshore structure costs were originally only based on the turbine rating but now also consider water depth. At depths of up to 25 m, the model assumes monopile structures. The model switches to jacket structures at depths between 25–60 m as the more cost-efficient option (Bosch et al., 2019). At depths between 60–1,000 m, the model assumes floating, semi-submersible structures (Stehly et al., 2020). We use these thresholds based on literature, but we acknowledge that they shift with the state of the art as monopoles can be deployed at depths of up to 40 m nowadays (Steelwind Nordenham, 2020). Power transmission costs are originally based on the turbine rating, but now they also consider the distance from the wind farm to the onshore connection point. At distances of up to 50 km, HVAC cables are used, and at further distances, HVDC cables are used. Furthermore, transportation, port and staging equipment, and installation cost originally footed on the turbine rating. Here, they are summarised under one cost component and calculated on a per-turbine basis (Bosch et al., 2019; Myhr et al., 2014) with most recent industry data (Stehly et al., 2020).

Second, we calibrate the cost model with technology-specific correction factors derived from the most recent cost review report by NREL (Stehly et al., 2020). We believe that the location- and technology-specific costs for fixed-bottom and floating wind farms can be adequately estimated with these modifications. Table S2 shows the original cost functions and all modifications.

#### Grid connection and local electricity tariffs

We connect wind farms either to Indonesian cities of the varying administrative levels or substations at 70 kV and above. From a private perspective, it would be reasonable to exclude off-grid areas, as it is not the responsibility of wind farm developers to build and maintain public grid infrastructure. Nonetheless, we still include them to reveal interesting locations for national grid extension and rural electrification.

The local electricity tariff can be assigned once a wind farm is connected to a city or substation. In Indonesia, the tariff for renewable electricity production is based on Power Purchase Agreements (PPA) between the power plant operator and Indonesia’s state-owned utility company *Perusahaan Listrik Negara (PLN)*. The maximum receivable tariff is capped by the *Biaya Pokok Penyediaan (BPP – Basic cost of electricity provision)*. The BPP reflects the electricity generation costs and is calculated for regions and the entire country. If the regional BPP is higher than the national BPP, a wind farm operator may receive up to 85% of the regional BPP. If the national BPP is higher than the regional BPP, the maximum receivable tariff is based on business-to-business negotiations. Since the details of the PPA are confidential, there is no reliable data on currently viable tariffs. Therefore, we assume that all wind farms receive 85% of the regional BPP. The set of regional BPP of 2018 ranged between 6.91–21.34 US¢(2018)/kWh (Ministry of Energy and Mineral Resources, 2019b), leading to receivable tariffs of 6.20–19.14 US¢(2021)/kWh depending on the location.

With the regional electricity tariffs, the economic wind potential is the aggregated rated power of all wind farms with an LCOE equal to or below the local electricity tariff. Although useful for this study, a limitation of this approach is that the 85% of regional BPP only serve as a price cap and depending on the negotiations with PLN, and the actual receivable tariff might vary. Moreover, Indonesia’s renewable energy policies undergo frequent reforms (Setyowati, 2020), and it is unclear whether and how long the current PPA scheme will exist.

#### Sensitivity analysis

To address the limitations elaborated above, we conduct a sensitivity analysis to understand their impact on the results better. First, we study how changes in site selection criteria affect the average LCOE, technical potential, and economic potential. We also add a carbon tax to the electricity tariffs to see how the economic potential per turbine changes. Second, we vary the representative model by ±20% to show the change of average LCOE, technical potential, and economic potential. The studied parameters are CAPEX, OPEX, wind speed, discount rate, hub height, and total efficiency including availability factor, as well as transmission and wake losses. For the adjustment of the wind speed for varying hub heights, we again use the power law as described earlier.

## Data Availability

• All data reported in this paper will be shared by the lead contact upon request • The code and key output data are publicly available via the 4TU research data repository under the https://doi.org/10.4121/19625259. • Any additional information required to reanalyze the data reported in this paper is available from the lead contact upon request.

## Supplemental information

Document S1. Tables S1 and S2

Table S3. Microsoft Excel file to calculate the impact of carbon tax on electricity tariff, related to STAR methods Input data taken from (Badan Pusat Statistik, 2019) for primary fossil energy consumption, from (Badan Pusat Statistik, 2019) for electricity related statistics, and from (Blok and Nieuwlaar, 2021) for emission factors.
