# Supplementary material for: How offshore wind could become economically attractive in low-resource regions like Indonesia
Source: iScience. 2022 Aug 16;25(9):104945. doi: 10.1016/j.isci.2022.104945 (PMC9442366; doi:10.1016/j.isci.2022.104945)
Supplement: Document S1. Tables S1 and S2 [file mmc1.pdf]

iScience, Volume *25*

## Supplemental information

# How offshore wind could become economically attractive in low-resource regions like Indonesia

**Jannis Langer, Sergio Simanjuntak, Stefan Pfenninger, Antonio Jarquin Laguna, George Lavidas, Henk Polinder, Jaco Quist, Harkunti Pertiwi Rahayu, and Kornelis Blok**

# Supplementary Tables

*Supplementary Table 1. Currency conversion rates. Conversion from EUR(year) to USD(year) based on* (Macrotrends, 2021). *Conversion from USD(year) to USD(2021) based on* (Bureau of Labour Statistics, 2021). *Related to STAR Methods.*

| **Year** | **EUR(year) to USD(year)** | **USD(year) to USD(2021)** |
|---|---|---|
| 2021 | 1.2 | 1 |
| 2020 | 1.14 | 1.01 |
| 2019 | 1.12 | 1.04 |
| 2018 | 1.18 | 1.06 |
| 2017 | 1.13 | 1.08 |
| 2016 | 1.11 | 1.10 |
| 2015 | 1.11 | 1.12 |
| 2014 | 1.33 | 1.12 |
| 2013 | 1.33 | 1.14 |
| 2012 | 1.29 | 1.15 |
| 2011 | 1.39 | 1.19 |
| 2010 | 1.33 | 1.21 |
| 2009 | 1.39 | 1.24 |
| 2008 | - | 1.24 |
| 2007 | - | 1.29 |
| 2006 | - | 1.32 |
| 2005 | - | 1.37 |
| 2004 | - | 1.41 |
| 2003 | - | 1.44 |
| 2002 | - | 1.48 |

*Supplementary Table 2. Turbine cost model used in this study. For the wind farm cost, the total turbine cost are multiplied with the number of turbines of the wind farm. For currency adjustment, the conversion rates in Supplementary Table 1 are used. If not stated otherwise, the cost functions are based on* (Fingersh et al., 2006). *Related to STAR Methods.*

| **Component** | **Cost function based on** | **Currency-adjusted cost function in US$(2021)** | **Remark** |
|---|---|---|---|
| Blades | Rotor radius *d/2* | $\frac{\left(\left(0.4019*\left(\frac{d}{2}\right)^{3}-955.24\right)+2.7445*\left(\frac{d}{2}\right)^{2.5025}\right)*3*1.48}{1-0.28}$ | - |
| Hub | Rotor diameter $d$, mass of a blade $m_{blade}$ and hub $m_{hub}$ <br> $m_{blade}=\left(0.1452*\left(\frac{d}{2}\right)^{2.9158}\right)$ <br> $m_{hub}=0.954*m_{blade}+5{,}680.3$ | $m_{hub}*4.25*1.48$ | - |
| Pitch | Rotor diameter $d$ | $2.28*(0.2106*d^{2.6578})*1.48$ | - |
| Cone | Rotor diameter $d$ and mass of cone $m_{cone}$ <br> $m_{cone}=18.5*d-520.5$ | $m_{cone}*5.57*1.48$ | - |
| Low-Speed Shaft | Rotor diameter $d$ | $0.1*d^{2.887}*1.48$ | Original factor 0.01 does not match with costs in example calculation in (Fingersh et al., 2006), hence adjustment to 0.1. |
| Bearing | Rotor diameter $d$ and mass of bearing $m_{bearing}$ <br> $m_{bearing}=\left(d*\frac{8}{600}-0.033\right)*0.0092*d^{2.5}$ | $2*m_{bearing}*17.6*1.48$ | - |
| Gearbox | Type of drivetrain and rated power $P_{rated}$ | **Direct drive:** 0 <br> **Three-stage planetary/ helical:** <br> $16.45*P_{rated}^{1.249}*1.48$ | - |
| Generator | Type of drivetrain and rated power $P_{rated}$ | **Direct drive:** <br> $P_{rated}*219.33*1.48$ <br> **Three-stage planetary/ helical:** <br> $P_{rated}*65*1.48$ | - |

*(continued)*

| Component | Cost function based on | Currency-adjusted cost function in US$(2021) | Remark |
|---|---|---|---|
| Mainframe | Type of drivetrain and rotor diameter *d* | **Direct drive:**<br>$1.96 * 627.28 * d^{0.85} * 1.48$<br>**Three-stage planetary/ helical:**<br>$1.96 * 9.489 * d^{1.953} * 1.48$ | Factor 1.96 added due to discrepancies with the example calculation in (Fingersh et al., 2006). |
| Brake | Rated power $P_{rated}$ | $(1.9894 * P_{rated} - 0.1141) * 1.48$ | - |
| Variable-Speed Electronics | Rated power $P_{rated}$ | $79 * P_{rated} * 1.48$ | - |
| Yaw | Rotor diameter *d* | $2 * (0.0339 * d^{2.964}) * 1.48$ | - |
| Platform | Type of drivetrain, mass of mainframe $m_{main}$ and rotor diameter *d*<br>**Direct drive:**<br>$m_{main} = 1.96 * 1.228 * d^{1.953}$<br>**Three-stage planetary/ helical:**<br>$m_{main} = 1.96 * 2.233 * d^{1.953}$<br><br>$m_{platform} = 0.125 * m_{main}$ | $8.7 * m_{platform} * 1.48$ | Factor 1.96 added due to discrepancies with the example calculation in (Fingersh et al., 2006). |
| Electronic Connections | Rated power $P_{rated}$ | $40 * P_{rated} * 1.48$ | - |
| Hydraulics Cooling | Rated power $P_{rated}$ | $12 * P_{rated} * 1.48$ | - |
| Nacelle | Rated power $P_{rated}$ | $11.537 * P_{rated} + 3{,}849.7 * 1.48$ | - |
| Control, Safety & Monitoring | Constant | $55{,}550 * 1.48$ | - |
| Tower | Mass of tower $m_{tower}$, rotor diameter *d* and hub height *h*<br>$m_{tower} = 0.3973 * \frac{\pi}{4} * d^2 * h - 1{,}414$ | $1.5 * m_{tower} * 1.48$ | - |
| Marinisation | Turbine and tower cost $C_{turb\&tower}$ | $0.135 * C_{turb\&tower}$ | - |
| Turbine and Tower Cost | - | Sum of all components above multiplied with correction factor *X* = 0.81 | Correction factor *X* based on cost developments reported in (Stehly et al., 2020) in US$(2019). |

*(continued)*

| Component | Cost function based on | Currency-adjusted cost function in US$(2021) | Remark |
|---|---|---|---|
| Offshore Structure | Water depth $D$ and rated power $P_{rated}$ | **If $D \leq 25$ m → Monopile**<br>$(0.201 * D^2 + 0.613 * D + 411.464) * P_{rated} * 1.11 * 1.10$<br>**If 25 m < $D \leq 55$ m → Jacket**<br>$(0.114 * D^2 - 2.270 * D + 531.738) * P_{rated} * 1.11 * 1.10$<br>**If 55 m < $D \leq 1{,}000$ m → Floating**<br>$(0.774 * D + 680.651) * P_{rated} * 1.11 * 1.10$ | Cost function based on (Bosch et al., 2019), because the cost function in (Fingersh et al., 2006) was not depth-dependent, which necessitated the use of a modified cost function as shown to the left. |
| Power Transmission | Distance to onshore connection $l$ and rated power $P_{rated}$ | **If $l \leq 50$ km → HVAC**<br>$(8.5 * l + 56.8) * P_{rated} * 1.11 * 1.10$<br>**If l > 50 km → HVDC**<br>$(2.2 * l + 387.8) * P_{rated} * 1.11 * 1.10$ | Cost function based on (Bosch et al., 2019), because the cost function in (Fingersh et al., 2006) was not distance-dependent, which necessitated the use of a modified cost function as shown to the left. |
| Permits & Engineering | Rated power $P_{rated}$ | **Fixed-bottom turbines:**<br>$208 * P_{rated} * 1.04$<br>**Floating turbines:**<br>$250 * P_{rated} * 1.04$ | Modified function based on cost developments reported in (Stehly et al., 2020) in US$(2019). |
| Personnel Equipment | Constant | $60{,}000 * 1.44$ | - |
| Scour Protection | Rated power $P_{rated}$ | $55 * P_{rated} * 1.44$ | - |
| Port Staging, Transport & Installation | Constant | **Fixed-bottom turbines:**<br>$2{,}688{,}000 * 1.04$<br>**Floating turbines:**<br>$1{,}212{,}000 * 1.04$ | Summarises the costs of port staging, transport, installation in (Fingersh et al., 2006). The costs apply on a per-turbine basis to remove the bias for the rated power in (Fingersh et al., 2006). The used values are based on the cost reported in (Stehly et al., 2020). |
| Balance of System Cost | - | Sum of costs for structure, transmission, permits & engineering, personnel equipment, scour protection, and staging, transport & installation | |
| Soft Costs | Rated power $P_{rated}$ | **Fixed-bottom turbines:**<br>$733 * P_{rated} * 1.04$<br>**Floating turbines:**<br>$878 * P_{rated} * 1.04$ | Modified function based on cost developments reported in US$(2019). Includes commissioning, decommissioning, contingency, construction finance, and insurance (Stehly et al., 2020). |

*(continued)*

| **Component** | **Cost function based on** | **Currency-adjusted cost function in US$(2021)** | **Remark** |
|---|---|---|---|
| Variable OPEX | Annual electricity production $E_a$ (excluding efficiencies and availability factor) | $(0.02 + 0.00108) * 1.48 * E_a$ | Summarises costs for operation & maintenance and bottom lease. |
| Fixed OPEX | Rated power $P_{rated}$ | $17 * 1.48 * P_{rated}$ | - |
| Total OPEX | - | $(OPEX_{var} + OPEX_{fixed}) * X$<br>**Fixed-bottom turbines:** $X = 0.8$<br>**Floating turbines:** $X = 0.84$ | Correction factor $X$ based on cost developments reported in (Stehly et al., 2020) in US$(2019). For both variable and fixed OPEX, we do not differentiate between different drivetrains, which in practice might affect OPEX. |

# References

Bosch, J., Staffell, I., Hawkes, A.D., 2019. Global levelised cost of electricity from offshore wind. Energy 189. https://doi.org/10.1016/j.energy.2019.116357

Bureau of Labour Statistics, 2021. CPI Inflation Calculator [WWW Document]. URL https://data.bls.gov/cgi-bin/cpicalc.pl?cost1=1.00&year1=201801&year2=202101 (accessed 5.19.21).

Fingersh, L., Hand, M., Laxson, A., 2006. Wind Turbine Design Cost and Scaling Model, NREL.

Macrotrends, 2021. EUR USD - Historical Annual Data [WWW Document]. URL https://www.macrotrends.net/2548/euro-dollar-exchange-rate-historical-chart (accessed 9.16.21).

Stehly, T., Beiter, P., Duffy, P., 2020. 2019 Cost of Wind Energy Review, National Renewable Energy Laboratory. https://doi.org/NREL/TP-5000-78471
